# Using Operational Analysis to Improve Access to Pulmonary Function Testing

**DOI:** 10.1155/2016/5269374

**Published:** 2016-04-07

**Authors:** Ada Ip, Raymond Asamoah-Barnieh, Diane P. Bischak, Warren J. Davidson, W. Ward Flemons, Sachin R. Pendharkar

**Affiliations:** ^1^W21C Research and Innovation Centre, O'Brien Institute for Public Health, Cumming School of Medicine, University of Calgary, Calgary, AB, Canada T2N 4Z6; ^2^Operations and Supply Chain Management, Haskayne School of Business, University of Calgary, Calgary, AB, Canada T2N 1N4; ^3^Department of Medicine, Cumming School of Medicine, University of Calgary, Calgary, AB, Canada T2N 4Z6; ^4^Department of Community Health Sciences, Cumming School of Medicine, University of Calgary, Calgary, AB, Canada T2N 4Z6

## Abstract

*Background*. Timely pulmonary function testing is crucial to improving diagnosis and treatment of pulmonary diseases. Perceptions of poor access at an academic pulmonary function laboratory prompted analysis of system demand and capacity to identify factors contributing to poor access.* Methods*. Surveys and interviews identified stakeholder perspectives on operational processes and access challenges. Retrospective data on testing demand and resource capacity was analyzed to understand utilization of testing resources.* Results*. Qualitative analysis demonstrated that stakeholder groups had discrepant views on access and capacity in the laboratory. Mean daily resource utilization was 0.64 (SD 0.15), with monthly average utilization consistently less than 0.75. Reserved testing slots for subspecialty clinics were poorly utilized, leaving many testing slots unfilled. When subspecialty demand exceeded number of reserved slots, there was sufficient capacity in the pulmonary function schedule to accommodate added demand. Findings were shared with stakeholders and influenced scheduling process improvements.* Conclusion*. This study highlights the importance of operational data to identify causes of poor access, guide system decision-making, and determine effects of improvement initiatives in a variety of healthcare settings. Importantly, simple operational analysis can help to improve efficiency of health systems with little or no added financial investment.

## 1. Introduction

Respiratory diseases are common and are responsible for almost 400,000 deaths in the United States per year [1]. In addition, they have significant economic impacts on the American healthcare system accounting for $154 billion (USD) in direct and indirect costs [1]. Pulmonary function (PF) testing is an important tool to evaluate respiratory symptoms and manage respiratory diseases. In combination with clinical assessment, PF testing improves both the diagnostic accuracy and management of respiratory diseases.

Health systems are often challenged to provide timely access to important diagnostic services [2]. While insufficient capacity may play a role, variations in demand and capacity or inefficient resource use can limit access even when capacity is apparently sufficient [3]. Adding capacity is often suggested as the solution to long wait times for health services, usually at a significant cost. In contrast, access may be improved by process-based interventions designed to increase the efficiency of resource use. Foundational to this type of improvement activity is a detailed analysis of system performance, which can assist in identifying contributors to poor access, guide the selection of system changes, and facilitate the evaluation of improvement initiatives [4]. Without such a system perspective, attempts to improve access to health services are likely to be unsuccessful.

This study examined the lack of timely PF testing at a large teaching hospital, which had led to dissatisfaction among physicians, administrative staff, and patients. Physicians had begun to request testing at non-hospital based, independent PF laboratories, which was inconvenient for patients and led to delays in the availability of test results at the time of clinical assessment. While physician's offices perceived a lack of available testing slots, the PF laboratory staff noted that there were many unfilled slots, suggesting that laboratory capacity may in fact have been adequate to meet the demand for testing. A systems-based analysis was undertaken to understand this discrepancy and to identify additional contributors to poor access.

## 2. Methods

### 2.1. Study Design

The study was conducted in a tertiary care academic PF laboratory located at the Foothills Medical Centre (FMC) in Calgary, Canada. There were two components to this study. First, a qualitative analysis was conducted using data gathered from surveys and interviews. Second, a quantitative analysis was performed using retrospective data on testing demand and resource capacity from April 1, 2013, to December 31, 2013. Additional details on study methods are available in Supplementary Material available online at http://dx.doi.org/10.1155/2016/5269374.

The Conjoint Health Research Ethics Board at the University of Calgary approved this study (approval numbers E-25243 and REB13-0554).

### 2.2. Study Setting

The FMC PF laboratory provides a broad range of PF testing and conducts over 7000 tests annually. Testing is ordered by respirologists for both general respiratory clinics and subspecialty clinics (e.g., bronchiectasis, lung transplant) and is usually requested for the same day as the patient's clinic appointment. Scheduling has two steps: the clinic appointment is scheduled by the respirologist's office and PF tests are scheduled by the PF laboratory staff. Respirologists may also order PF tests independently of clinic appointments.

The FMC PF laboratory schedule is divided into generic and reserved blocks of testing time. Generic testing time is available to all ordering physicians, whereas subspecialty clinics have reserved testing time to ensure timely access to testing for patients attending these clinics. For example, a subspecialty clinic held on Wednesday may reserve six 15-minute testing time slots (90 minutes) for patients to be tested just before their clinic visit. Unused reserved slots are typically not released for use by other physicians. Subspecialty clinics are not required to use all reserved time slots before scheduling into generic time slots.

### 2.3. Data Collection

#### 2.3.1. Qualitative Data

An Internet-based survey was administered to laboratory staff, respirologists, and medical office assistants to gain insight into operational processes of the PF laboratory and identify areas that could be explored further with interviews. A research associate conducted individual semistructured interviews with these stakeholders to gather additional perspectives on barriers and improvements to access. Survey and interview questions are available in the online Supplementary Material. The analysis and results were presented to these stakeholder groups at a forum at the end of the study period.

#### 2.3.2. Testing Demand

Demand for PF testing was defined as the total number of minutes of testing scheduled on each day. This calculation included testing time allocations for patients who underwent testing, cancelled their appointment on the day of the appointment (same-day cancellation), or did not attend their appointment (no-shows). Same-day cancellations and no-shows were included because they used up available appointment time.

Demand for subspecialty clinics was calculated by collecting the number of minutes of testing scheduled per day for each clinic, including testing minutes scheduled within reserved slots and generic slots.

#### 2.3.3. Capacity

Pulmonary function testing is available during regular business hours on weekdays, with the exception of statutory holidays. Resource capacity was defined as the time available for testing. It was calculated by determining the total available testing time for each day of the week. Reserved capacity was calculated by determining the available testing time for each clinic that had reserved capacity.

### 2.4. Analysis

Analysis of interview data included coding, categorization by stakeholder group, and thematization [5, 6]. Data displays [5] were used to identify the main barriers to access and the main recommendations to improve access.

Demand and capacity data were used to calculate the utilization rate for PF laboratory resources over the study period. Daily resource utilization was calculated by dividing the total daily demand for testing by the total resource capacity on that day. Utilization rates were also calculated for reserved slots for each subspecialty clinic.

The primary outcome was the overall resource utilization of the PF laboratory over the study period. The secondary outcome was the resource utilization of the reserved time slots for each clinic. Analysis included descriptive statistics and graphical analysis to demonstrate individual utilization values and variation over time. Data are presented as mean (standard deviation) unless otherwise stated.

## 3. Results

### 3.1. Qualitative Analysis

Twenty-seven survey responses were received and 17 interviews were conducted.  Table 1 shows the breakdown of surveys and interviews by stakeholder group and suggests that a broad range of perspectives were obtained.

The qualitative analysis demonstrated conflicting perspectives among different stakeholder groups in regard to patient access. Generally, respiratory therapists and booking clerks who are physically located at the PF laboratory described access as “very good” and “improved.” However, some physicians and medical staff who are not physically located at the PF laboratory expressed difficulties with access.

### 3.2. PF Laboratory Utilization

Between April 1, 2013, and December 31, 2013, 3732 unique patients attended a total of 5184 appointments at the PF laboratory on 189 testing days. Of the 5184 appointments, 4811 (93%) were checked-in appointments, 263 (5%) were no-shows, and 110 (2%) were same-day cancellations. An additional 74 patients (1%) had testing at independent PF facilities.

**Figure 1:**
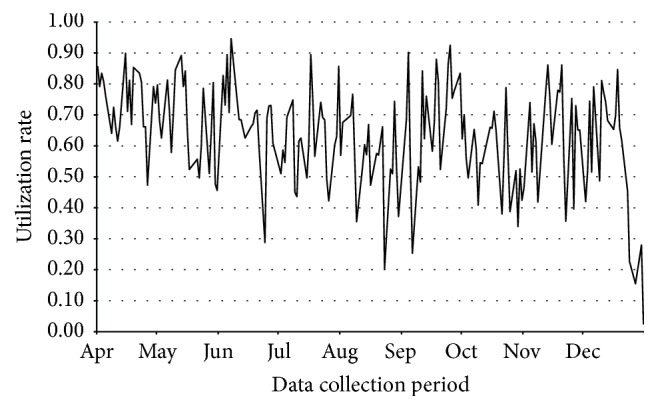
Daily utilization rates.

Figure 1 shows the daily utilization rate from April 1 to December 31, 2013; monthly demand, capacity, and average utilization rates are shown in Table 2. Daily resource capacity varied depending on the day of the week but remained the same across weeks, resulting in variability in monthly capacity (see Table E1 in the Supplementary Material online). A respiratory therapist position was added in October 2013, resulting in an increase in resource capacity. The overall mean daily resource utilization rate was 0.64 (0.15).

**Figure 2:**
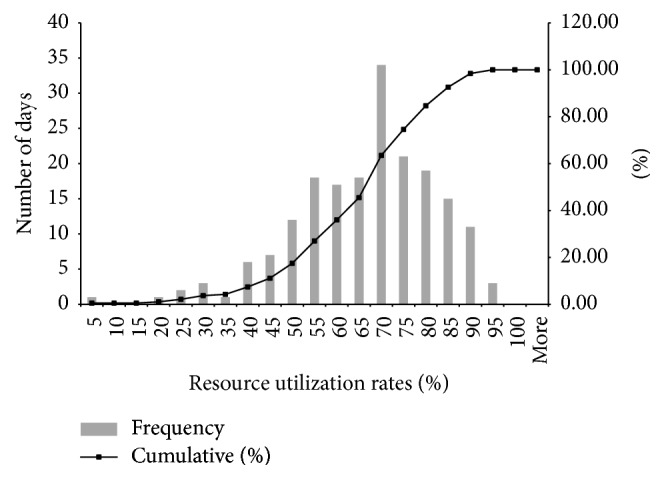
Histogram of daily resource utilization.

While Table 2 shows that monthly average utilization values consistently fell below 0.75, there was considerable variation in the daily demand and thus in the daily utilization of testing capacity.  Figure 2 reveals that the utilization was above 0.85 on only 7% of days. The highest observed utilization rate was 0.95.

Utilization rates were recalculated for October to December 2013 using resource capacity values from April to September, to determine whether lower utilization during the latter months was due to increased capacity or lower demand. The adjusted mean utilization values for October, November, and December were 0.58, 0.68, and 0.59, respectively, and utilization was greater than 0.85 on only 8% of days (data not shown). Thus, the lower utilization was due to both an increase in capacity and a decrease in demand.

### 3.3. Utilization of Reserved Subspecialty Slots

**Figure 3:**
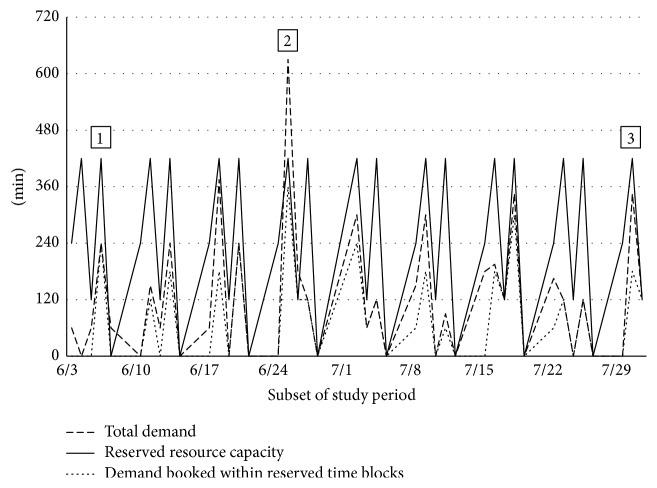
Subspecialty demand versus reserved resource capacity. Label 1: total subspecialty demand less than reserved capacity. Label 2: total subspecialty demand greater than reserved capacity. Label 3: subspecialty demand booked outside of reserved capacity.

Four subspecialty clinics had reserved slots in the scheduling system. The utilization of these reserved slots was consistently low, with the exception of one small subspecialty clinic with very consistent weekly demand. Data on demand, resource capacity, and use of reserved slots for a representative subspecialty clinic over eight weeks is presented in Figure 3. The mean utilization of reserved slots for this clinic was 0.41 (0.37).

Three common scenarios were identified for the utilization of reserved slots. There were 137 days (73%) where total subspecialty demand was less than reserved capacity (label 1). Unused capacity ranged from 15 to 420 minutes in a single day.

There were seven days (5%) where subspecialty demand exceeded the reserved resource capacity (label 2). However, on each of these days, there was adequate capacity within the rest of the laboratory schedule to meet the additional demand for testing.

At 47 days (25%), there was available reserved capacity but subspecialty demand was booked outside of the reserved capacity (label 3). The laboratory staff indicated that booking outside of the reserved slots occurred due to patient mobility issues, patient preferences, and attempts by laboratory clerical staff to reduce delays between PF testing and the patient's clinic appointment.

## 4. Discussion

This study demonstrated the use of simple operational data to understand the performance of a diagnostic testing facility. The results indicated that the FMC PF laboratory was underutilized despite the perception of poor access by some stakeholders. Furthermore, reserved slots for subspecialty clinics were poorly utilized, which often exacerbated the resource underutilization because subspecialty patients used generic slots while reserved slots could not be used by nonsubspecialty patients and thus remained unfilled.

It is known from operations research that variation in demand for system resources can adversely affect access to services. In healthcare settings, demand variation may be due to the number of patients requiring service or the services required by those patients. To counteract the negative implications of demand variation on system performance, a buffer of extra capacity is typically recommended, such that utilization does not exceed 0.85 [7, 8]. In this study, utilization levels were often well below this threshold, suggesting that any problems with access were due to inefficient processes rather than excessive demand or demand variation.

High no-show rates contribute to poor access in other healthcare settings, due to the inability to use scheduled appointment slots for other patients and the need to reschedule patients who have missed appointments [9, 10]. In the current study, no-shows and same-day cancellations totaled only 7% of all scheduled appointments and thus were not major contributors to unused capacity or the use of additional slots for rescheduling. Additionally, missed appointments and same-day cancellations were included in the demand estimate; since the utilization of PF laboratory resources remained below 0.85, the low utilization of PF testing capacity could not be attributed to these unfilled slots.

The reservation of appointment slots, known as “ring-fencing” capacity, is intended to provide better access to specific groups of patients, such as those deemed to be urgent. However, variation in demand could lead to unused capacity if reserved slots are not made available for general use. The result could paradoxically be an increase in queue length and wait times [3], as found in a number of clinical settings [11, 12]. In this study, the analysis of reserved slots illustrated three key findings. First, subspecialty demand was often much lower than the amount of reserved resource capacity, leading to the perception that demand for PF testing exceeded testing capacity since unused reserved time was inaccessible by general respiratory clinics. Second, when subspecialty demand exceeded reserved capacity, there was adequate resource capacity within the generic slots to accommodate the extra demand. Third, subspecialty tests were often scheduled outside of the reserved time, consuming generic time slots while reserved slots went unused.

Thus, this study suggests that access is not improved by cordoning off resources for subspecialty or urgent patients but rather by maintaining equitable availability across all patient groups. This approach reduces the administrative burden on clerical staff and eliminates the possibility that reserved resources will go unused. An alternate approach is to manage the variation in demand for testing directly rather than allocating PF testing capacity to different demand sources; for example, altering the schedule of clinics so that testing demand on each day of the week is similar would minimize the likelihood that the laboratory would be highly utilized on some days and poorly utilized on others.

This study also demonstrates the important role of operations research within healthcare systems. Simple operational data can provide valuable information to help understand system performance, evaluate system changes, and guide decision-making. In this study, operational data was used to explore the belief that the FMC PF laboratory had inadequate capacity to meet demand and that reserved slots would improve access. Prior to this analysis, administrative processes and capacity increases had been unnecessarily put in place to guarantee timely access. When presented with the results of this operational analysis, discussion among stakeholders guided the decision to eliminate ring-fencing for subspecialty clinics. Furthermore, the PF laboratory has also formed a quality committee comprised of key stakeholders. This committee will review demand, capacity, and utilization data on a regular basis to monitor system performance and adapt laboratory operations to optimize access. An example of the function of this committee would be to consider historical demand and utilization data before making changes to PF testing capacity.

A key finding of this project was the underutilization of testing slots. In general, utilization can be increased in two ways: by decreasing capacity or by increasing demand. Utilization was not expected to change simply by removing ring-fencing for subspecialty clinics, because this process change would not directly impact the total demand or total capacity at the laboratory. Without ring-fencing, however, utilization across appointment slots will become more uniform, and slots that had previously been reserved (and unused) will be available to any respirologist or subspecialty clinic. As the availability of slots becomes more customary and predictable, we expect that physicians will become more confident that their patients' testing at the laboratory will be completed on a timely basis. Consequently, over time less demand will be sent to non-hospital based independent facilities, and average utilization for the laboratory will increase. Given the likely delay between the implementation of these scheduling improvements and changes in physician practice, we were unable to confirm an increase in demand or utilization using system data. However, based on this analysis, the PF laboratory's quality committee will review this data at a later date to ensure that the expected improvements in system performance have occurred.

In some clinical areas, excess capacity is made available for unscheduled “walk-in” visits. While the PF laboratory already provides some unscheduled testing of hospital inpatients, additional allocation of testing resources to walk-in visits could impede access for patients who are attending a same-day respirologist appointment, depending on how these slots are made available. Based on this information, the decision was made to offer more scheduled testing to nonrespiratory clinics and other physicians (such as family physicians).

There are several limitations to the current study. First, the study was conducted at a tertiary academic centre, which may have different operational processes than nonacademic centres. However, we believe that the concept of using simple operational data to improve system performance is widely applicable to a variety of healthcare contexts. In most jurisdictions this data is often collected for administrative reasons and is relatively easy to analyze, making it interpretable by healthcare managers, administrative staff, and clinicians. Second, the causes of demand variation were not explored in this study. Since understanding and reducing variability are important steps to improving access, this issue has been identified as a focus for future study. Finally, data was from PF laboratory and clinical scheduling databases and was not collected for the purposes of this study. Although manual data entry may have resulted in administrative errors, we validated the data through regular meetings with the PF laboratory staff and manual chart reviews where required.

## 5. Conclusions

We have demonstrated that simple operational analysis can be used to improve the operations of PF laboratories. The insights from this study could be of use to other PF laboratories. First, demand, capacity, and scheduling data can help to confirm or refute stakeholder perceptions about access. Additionally, strategies to improve access may not achieve their intended outcome and may paradoxically worsen system performance. Finally, operational perspectives on system performance may help with resource allocation and scheduling decisions. Given the high prevalence of respiratory symptoms and the importance of PF testing in the management of respiratory disease, this type of analysis is crucial to improving the efficiency of care delivery.

## Supplementary Material

The Supplementary Material provides more information on the methods and results of the study. Appendix 1 and 2 consists of the survey and interview questions that were administered to physicians, laboratory staff and medical office assistants.

## Figures and Tables

**Table 1:** Survey and interview response rate for each stakeholder group.

Stakeholder group	Surveys (%)	Interviews (%)
Respirologists (*n* = 10)	10 (100)	6 (60)
Respiratory therapists (*n* = 10)	7 (70)	5 (50)
Administrative assistants (*n* = 12)	6 (50)	3 (25)
Other (*n* = 5)	4 (80)	3 (60)
Total (*n* = 37)	27 (73)	17 (46)

Other includes nurse practitioners and laboratory booking clerks.

**Table 2:** Monthly mean demand, capacity, and resource utilization rates.

	Demand (minutes)	Capacity (minutes)	Utilization, mean (SD)
April	32,985	44,480	0.74 (0.10)
May	29,505	43,795	0.67 (0.14)
June	27,885	40,160	0.69 (0.13)
July	27,540	44,390	0.62 (0.13)
August	23,670	41,800	0.57 (0.13)
September	28,110	40,160	0.70 (0.17)
October	26,040	46,590	0.56 (0.12)
November	27,360	42,105	0.65 (0.15)
December	23,610	43,020	0.58 (0.20)

## Additional Points

University of Calgary was the institution at which the work originated.
