# Supplementary material for: Using Operational Analysis to Improve Access to Pulmonary Function Testing
Source: Can Respir J. 2016 Apr 7;2016:5269374. doi: 10.1155/2016/5269374 (PMC4904535; doi:10.1155/2016/5269374)
Supplement: Supplementary file 1 — The Supplementary Material provides more information on the methods and results of the study. Appendix 1 and 2 consists of the survey and interview questions that were administered to physicians, laboratory staff and medical office assistants. [file 5269374.f1.docx]

# Supplementary Material

# Methods

## Study setting

PF testing can also be requested by community physicians and for hospital inpatients and will be accommodated if there are open slots in the scheduling system. The amount of time scheduled for PF testing for a given patient depends on the type of testing ordered. For example, basic spirometry is allocated 30 minutes, whereas a complete PF test consisting of spirometry and measurement of lung volumes and diffusion capacity for carbon monoxide is allocated 60 minutes.

Data Collection

### Qualitative Data

An internet-based survey was administered to laboratory staff, respirologists and medical office assistants, followed by individual semi-structured interviews with these stakeholders to gather additional perspectives. Survey and interview questions are available in the online supplementary material (See Appendix 1 and 2, below). Patients completed a separate questionnaire that explored preferences regarding waiting times for testing and between test and appointment times (data not shown).

### Testing Demand

Demand for testing for patients tested at the FMC PF laboratory during the data collection period was extracted from the PF laboratory’s scheduling system and the respirology clinic’s scheduling system. Key data elements extracted from each appointment included the PF appointment date, the requesting physician clinic (categorized either as a subspecialty clinic or general respirology) and the duration of the PF appointment based on the type of testing requested. Data that was unavailable from the electronic databases was acquired through manual chart review.

If testing could not be completed in a timely manner, respirologists occasionally ordered PF testing to be performed at independent PF laboratories that are publicly funded but privately administered in non-hospital settings. These tests were also included in the calculation of testing demand, since it was expected that these testing referrals would be repatriated to the FMC PF laboratory if access improved. Demand for tests administered in independent facilities was gathered manually via clerks in the respiratory and subspecialty clinics by asking arriving patients if they had their testing at another facility in advance of their clinic appointment.

### Resource Capacity

The PF laboratory equipment resources include four constant-volume body plethysmographs and two spirometers. Resource capacity data was extracted from the pulmonary function laboratory’s scheduling system. Daily resource capacity varied depending on the day of the week but remained the same across weeks. Resource capacity was modified to account for statutory holidays on which days the PF laboratory was closed.

### Analysis

An additional secondary outcome included the utilization of PF testing resources on each day of the week. Daily utilization rates were used to calculate monthly averages and averages based on the day of the week.

# Results

When categorized by day of the week, Table E1 shows that the average utilization rate was highest on Wednesday and lowest on Friday. In addition, Wednesday had the highest percentage of occurrences where utilization exceeded 0.85; however, only 5% had a utilization rate over 0.90. It was observed that more respiratory clinics were held on Wednesdays than any other day of the week.

Table E1. Mean Resource Utilization for Each Weekday.

|  | Demand | Capacity | Utilization,  Mean (SD) | Percentages of occurrences where utilization > 0.85 | Percentages of occurrences where utilization >0.90 |
| --- | --- | --- | --- | --- | --- |
| Monday | 46,410 | 72,705 | 0.64 (0.17) | 9% | 3% |
| Tuesday | 56,475 | 91,500 | 0.63 (0.14) | 3% | 0% |
| Wednesday | 55,935 | 76,530 | 0.73 (0.13) | 21% | 5% |
| Thursday | 47,250 | 74,460 | 0.64 (0.09) | 0% | 0% |
| Friday | 40,635 | 71,305 | 0.57 (0.18) | 3% | 3% |

# Appendix 1 – Online Survey

**Introduction**: This survey is about the Foothills Medical Center (FMC) Pulmonary Function Testing (PFT) facility.

1. Please indicate your role:

☐ Physician
☐ Administrative Assistant
☐ Secretary/Booking Clerk
☐ Laboratory Staff
☐ Other

1. For how many years have you been working with/at the FMC PF lab? __________
2. The Alberta Health Services vision defines Access, Quality and Sustainability as below:

- Access: Health services are obtained in the most suitable setting in a reasonable time and distance.
- Quality: Health services are effective in outcomes, safe, appropriate to user needs, acceptable to users and efficient in resource utilization.
- Sustainability: Health care must be delivered in a manner which is sustainable for the future and within available resources (including funding and people).

How, in general terms, should we be evaluating success in the lab with respect to the following:

| **Access**  **(e.g. Wait times)** | **Quality**  **(e.g. Meeting ATS standards, Patient provider satisfaction)** | **Sustainability**  **(e.g. Appropriate use of testing)** |
| --- | --- | --- |
|  |  |  |

1. Could you please suggest specific measures of FMC PFT Lab success in terms of:

| **AHS Goal** | **Strategic Dimensions** | **Measure/Metric** |
| --- | --- | --- |
| Quality | **Acceptability** – Health services are respectful and responsive to user needs, preferences and expectations (e.g. patient satisfaction) |  |
|  | **Appropriateness** – Health services are relevant to user needs and are based on accepted or evidence-based practice (e.g. clinical need for testing) |  |
|  | **Effectiveness** – Health services are provided based on scientific knowledge to achieve desired outcomes (e.g. test quality) |  |
|  | **Efficiency** – Resources are optimally used in achieving desired outcomes (e.g. ease of booking a test) |  |
|  | **Safety** – mitigate risks to avoid unintended or harmful results (e.g. testing of infectious patients) |  |
| Access | Appropriate health care services are available (e.g. wait time for test) |  |
| Sustainability | Resources are used in the most effective and efficient way (e.g. cost of repeated testing) |  |

1. On a scale of 1-5 with **5 being very important** and **1 being least important**, how do you rate the following FMC PFT Lab measures as measures of FMC PFT Lab success? Please check N/A if not applicable.

| AHS Goal | Measure/Metric | Importance Rating  1 2 3 4 5 N/A |
| --- | --- | --- |
| Quality | 1. Patient Satisfaction 2. Provider Satisfaction 3. Number of tests per patient per year 4. Percentage of tests that meet ATS standards 5. Percent utilization of testing machines (i.e. % of testing slots that are used). 6. Number of patients arriving at the lab without a requisition 7. No show rates 8. Rate of test cancellation by patients 9. Number of tests not completed prior to the outpatient clinic appointment | 1.       2.       3.       4.       5.       6.       7.       8.       9.       |
| Access | 1. Wait time between request for testing and appointment for testing 2. Wait time between completion of test and clinic appointment time 3. Ease of booking test | 1.       2.       3.       |
| Sustainability | 1. Overtime of lab staff 2. Cost of repeat testing | 1.       2.       |

1. What is the maximum number of days that a PFT could be performed prior to a clinic appointment and still be clinically useful?
2. Regarding the FMC PFT Lab, what do you believe that patients perceive as important for access to testing?
3. Regarding problems at the FMC PFT Lab:
   1. Could you identify some problems and barriers to access at the FMC PFT Lab?
   2. Could you suggest how the problems you have just identified could be solved?
4. Is there any extra information you would like to add regarding PFT at FMC?

# Appendix 2 – Interview Questions

## Control Question:

1. Briefly, what is your educational background and work experience?

## Main Questions

1. Could you tell your experience with PFT access?

- Probe: The “Why” of perceptions and preferences.

1. If PFT performance were to be measured, could you tell from your perspective, what could be good indicators of PFT performance on access?

- Probe: The Reason’s for the choice of indicators.

1. How well does the PFT laboratory currently perform on the indicators you have suggested?
2. Could you tell why the PFT lab is not able to get PF test results out exactly when the physicians need them? Please emphasize the root causes of the problem.
3. From your perspective, what process improvements might improve PFT performance on access?
4. Is there anything else you would like to add before this interview session closes?
